# Fast phylogenetic inference from typing data

**DOI:** 10.1186/s13015-017-0119-7

**Published:** 2018-02-15

**Authors:** João A. Carriço, Maxime Crochemore, Alexandre P. Francisco, Solon P. Pissis, Bruno Ribeiro-Gonçalves, Cátia Vaz

**Affiliations:** 1 Faculdade de Medicina, Instituto de Microbiologia and Instituto de Medicina Molecular, Universidade de Lisboa, Lisboa, Portugal; 2 Department of Informatics, King’s College London, London, UK; 3 INESC-ID Lisboa, Rua Alves Redol 9, 1000-029 Lisboa, Portugal; 4 Instituto Superior Técnico, Universidade de Lisboa, Lisboa, Portugal; 5 Instituto Superior de Engenharia de Lisboa, Instituto Politécnico de Lisboa, Lisboa, Portugal

**Keywords:** Computational biology, Phylogenetic inference, Hamming distance

## Abstract

**Background:**

Microbial typing methods are commonly used to study the relatedness of bacterial strains. Sequence-based typing methods are a gold standard for epidemiological surveillance due to the inherent portability of sequence and allelic profile data, fast analysis times and their capacity to create common nomenclatures for strains or clones. This led to development of several novel methods and several databases being made available for many microbial species. With the mainstream use of High Throughput Sequencing, the amount of data being accumulated in these databases is huge, storing thousands of different profiles. On the other hand, computing genetic evolutionary distances among a set of typing profiles or taxa dominates the running time of many phylogenetic inference methods. It is important also to note that most of genetic evolution distance definitions rely, even if indirectly, on computing the pairwise Hamming distance among sequences or profiles.

**Results:**

We propose here an average-case linear-time algorithm to compute pairwise Hamming distances among a set of taxa under a given Hamming distance threshold. This article includes both a theoretical analysis and extensive experimental results concerning the proposed algorithm. We further show how this algorithm can be successfully integrated into a well known phylogenetic inference method, and how it can be used to speedup querying local phylogenetic patterns over large typing databases.

## Background

### Introduction

The evolutionary relationships between different species or *taxa* are usually inferred through known phylogenetic analysis techniques. Some of these techniques rely on the inference of phylogenetic trees, which can be computed from DNA or Protein sequences, or from allelic profiles where the sequences of defined loci are abstracted to categorical indexes. The most popular method is MultiLocus sequence typing (MLST) [1] that typically uses seven 450–700 bp fragments of housekeeping genes for a given species. Phylogenetic trees are also used in other contexts, such as to understand the evolutionary history of gene families, to allow phylogenetic foot-printing, to trace the origin and transmission of infectious diseases, or to study the co-evolution of hosts and parasites [2, 3].

In traditional phylogenetic methods, the process of phylogenetic inference starts with a multiple alignment of the sequences under study that is then corrected using models of DNA or Protein evolution. Tree-building methodologies can then be applied on the resulting distance matrix. These methods rely on some distance-based analysis of sequences or profiles [4].

Distance-based methods for phylogenetic analysis rely on a measure of genetic evolution distance, which is often defined directly or indirectly from the fraction of mismatches at aligned positions, with gaps either ignored or counted as mismatches. A first step of these methods is to compute this distance between all pairs of sequences. The simplest approach is to use the Hamming distance, also known as observed *p*-distance, defined as the number of positions at which two aligned sequences differ. Note that the Hamming distance between two sequences underestimates their true evolutionary distance and, thus, a correction formula based on some model of evolution is often used [2, 4]. Although distance-based methods not always produce the best tree for the data, usually they also incorporate an optimality criterion into the distance model for getting more plausible phylogenetic reconstructions, such as the minimum evolution criterion [5], the least squares criterion [6] or the clonal complexes expansion and diversification [7]. Nevertheless, this category of methods are much faster than Maximum likelihood or Bayesian inference methods [8], making them excellent choices for the primary analysis of large data sets.

**Fig. 1:**
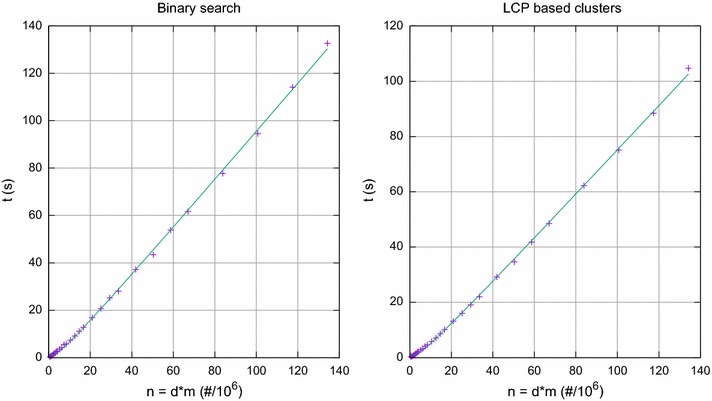
Synthetic datasets, with $\sigma =2$ and $k=\lfloor m/(2\log m)\rfloor$ according to Theorem 1. Running time for computing pairwise distances by finding lower and higher bounds in the SA, and by processing LCP based clusters, as function of the input size $n=dm$

**Fig. 2:**
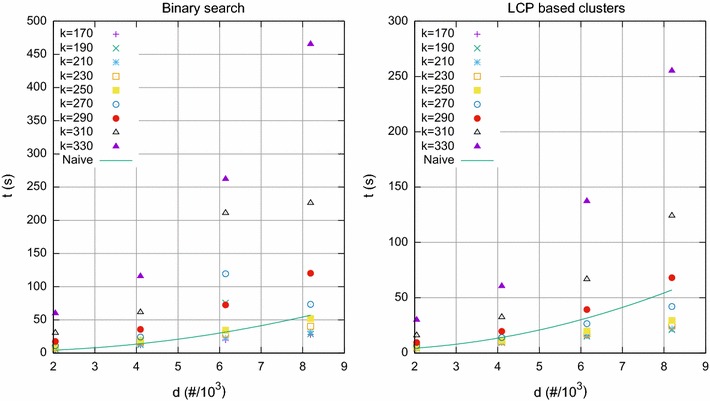
Synthetic datasets, with $\sigma =2$ and $m=4096$. Running time for computing pairwise distances by finding lower and higher bounds in the SA, and by processing LCP based clusters, as function of the number *d* of profiles and for different values of *k*

**Fig. 3:**
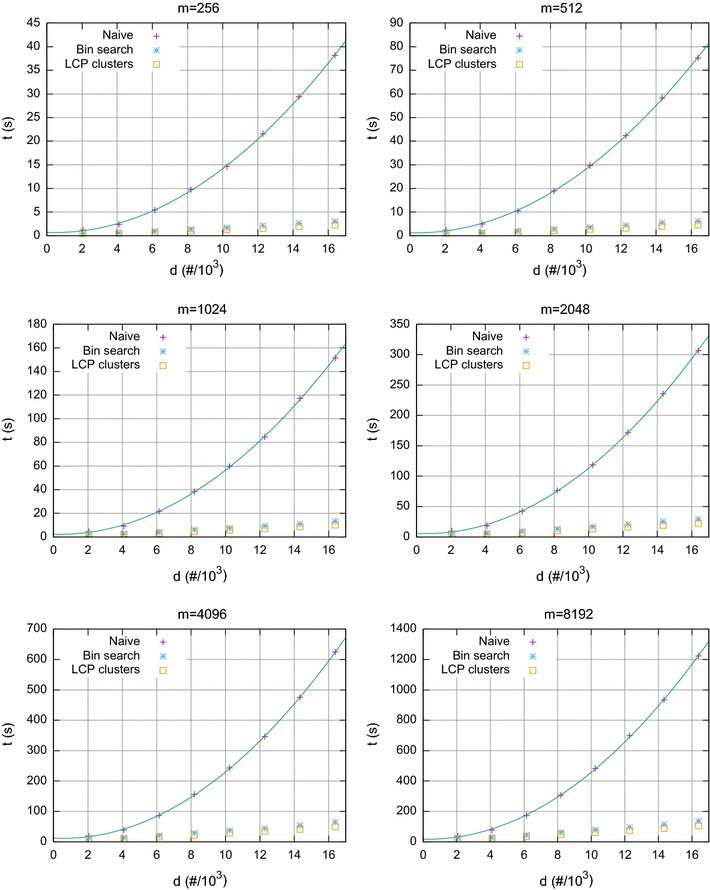
Synthetic datasets, with $\sigma =2$ and $k=\lfloor m/(2\log m)\rfloor$ according to Theorem 1. Running time for computing pairwise distances naïvely, by finding lower and higher bounds in the SA, and by processing LCP based clusters, as a function of the number *d* of profiles

**Fig. 4:**
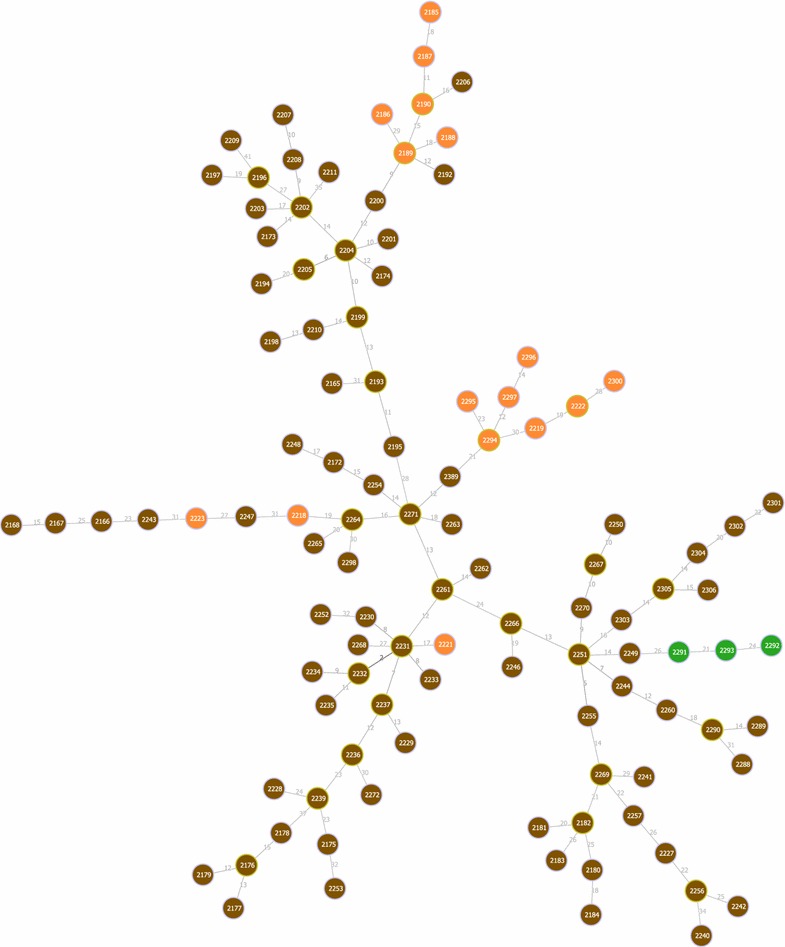
The tree inferred for the largest connected component found with $k=52$ for the *C. jejuni* dataset. Image produced by PHYLOViZ [35]

Most of the distance-based methods are agglomerative methods. They start with each sequence being a singleton cluster and, at each step, they join two clusters. The iterative process stops when all sequences are part of a single cluster, resulting in a phylogenetic tree. At each step the candidate pair is selected taking into account the distance among clusters as well as the optimality criterion chosen to adjust it.

The computation of a distance matrix (2D array containing the pairwise distances between the elements of a set) is a common first step for distance-based methods, such as eBURST [9], goeBURST [10], Neighbor Joining [11] and UPGMA [12]. This particular step dominates the running time of most methods, taking $\Theta (m d^2)$ time in general, *d* being the number of sequences or profiles and *m* the length of each sequence or profile. For large-scale datasets this running time may be quite problematic. And nowadays, with the mainstream use of High Throughput Sequencing, the amount of data being accumulated in typing databases is huge. It is common to find databases storing thousands of different profiles for a single microbial species, with each profile having thousands of loci [13, 14].

**Table 1:** Data structures used in our approach for each step

Profile indexing	Candidate profile pairs enumeration	Pairs verification
Suffix array	Binary search	Naïve
	LCP based clusters	$\text {RMQ}_\text {LCP}$

However, depending on application, on the underlying model of evolution and on the optimality criterion, it may not be strictly necessary to be aware of the complete distance matrix. There are methods that continue to provide optimal solutions without a complete matrix. For such methods, one may still consider a truncated distance matrix and several heuristics, combined with final local searches through topology rearrangements, to improve the running time [6]. The goeBURST algorithm, one of our use cases in this article, is an example of a method that can work with truncated distance matrices by construction, i.e., one needs only to know which pairs are at Hamming distance at most *k*.

### Our results

We propose here an average-case $\mathcal {O}(m d)$-time and $\mathcal {O}(m d)$-space algorithm to compute the pairs of sequences, among *d* sequences of length *m*, that are at distance at most *k*, when $k < \frac{(m-k-1) \cdot \log \sigma }{\log m d}$, where $\sigma$ is the size of the sequences alphabet. We support our result with both a theoretical analysis and an experimental evaluation on synthetic and real datasets of different data types (MLST, cgMLST, wgMLST and SNP). We further show that our method improves goeBURST, and that we can use it to speedup querying local phylogenetic patterns over large typing databases.

A preliminary version of this paper was presented at the Workshop on Algorithms in Bioinformatics (WABI) 2017 [15].

## Methods

### Closest pairs in linear time

Let *P* be the set of profiles (or sequences) each of length *m*, defined over an integer alphabet $\Sigma$, (i.e., $\Sigma = \{1,\ldots ,m^{O(1)}\}$), with $d=|P|$ and $\sigma =|\Sigma |$. Let also $H:P \times P \rightarrow \{0,\ldots ,m\}$ be the function such that *H*(*u*, *v*) is the Hamming distance between profiles $u,v\in P$. Given an integer threshold $0< k < m$, the problem is to compute all pairs $u,v\in P$ such that $H(u,v) \le k$, and the corresponding *H*(*u*, *v*) value, faster than the $\Theta (m d^2)$ time required to compute naïvely the complete distance matrix for the *d* profiles of length *m*.

**Table 2:** Real datasets used in the experimental evaluation

Dataset	Typing method	Profile length	Number of distinct elements	References
*Campylobacter jejuni*	wgMLST	5446	5669	(*)
*Salmonella enterica*	wgMLST	3002	6861	[13]
*Salmonella typhi*	SNP	22,143	1534	[36]
*Streptococcus pneumoniae*	cgMLST	235	1968	[37–39]

(*) Dataset provided by the Molecular Microbiology and Infection Unit, IMM

We address this problem by indexing all profiles *P* using the suffix array (denoted by SA) and the longest common prefix (denoted by LCP) array [16]. We rely also on a range minimum queries (RMQ) data structure [17, 18] over the LCP array (denoted by $\text {RMQ}_\text {LCP}$). The problem is then solved in three main steps:

1. Index all profiles using the SA data structure.
2. Enumerate all candidate profile pairs given the maximum Hamming distance *k*.
3. Verify each candidate profile pair by checking if the associated Hamming distance is no more than *k*.

Table 1 summarizes the data structures and strategies followed in each step. Profiles are concatenated and indexed using SA. Depending on the strategy to be used, we further process the SA and build the LCP array and pre-process it for fast RMQ. This allows for enumerating candidate profile pairs and computing distances faster. In what follows, we detail the above steps and show how the data structures are used to improve the overall running time.

#### Step 1: Profile indexing

Profiles are concatenated and indexed in an SA in $\mathcal {O}(m d)$ time and space [19, 20]. Let us denote this string by *s*. Since we only need to compute the distances between profiles that are at Hamming distance at most *k*, we can conceptually split each profile into *k* non-overlapping *blocks* of length $\mathcal{L}= {\lfloor \tfrac{m}{k+1} \rfloor }$ each. It is then folklore knowledge that if two profiles are within distance *k*, they must share at least one such block of length $\mathcal{L}$. Our approach is based on using the SA of *s* to efficiently identify matching blocks among profile pairs. This lets us quickly filter in candidate profile pairs and filter out the ones that can never be part of the output.

#### Step 2: Candidate profile pairs enumeration

The candidate profile pairs enumeration step provides the pairs of profiles that do not differ in more than *k* positions, but it may include spurious pairs. Since SA is an ordered structure, a simple solution is to use a binary search approach. For each block of each profile, we can obtain in $$\mathcal {O}(\mathcal{L} \log \ n)$$ time, where $$ n = m d$$, all the suffixes that have that block as a prefix. If a given match is not aligned with the initial block, i.e., it does not occur at the same position in the respective profile, then it should be discarded. Otherwise, a candidate profile pair is reported. This searching procedure is done in $$\mathcal {O}(d k \mathcal{L} \log n) = \mathcal {O}(n \log n)$$ time.

Another solution relies on computing the LCP array: the longest common prefix between each pair of consecutive elements within the SA. This information can also be computed in $\mathcal {O}(n)$ time and space [21]. Since SA is an ordered structure, for the contiguous suffixes $s_{i}, s_{i+1}, s_{i+2}$ of *s*, with $0 \le i< n-2$, we have that the common prefix between $s_{i}$ and $s_{i+1}$ is at least as long as the common prefix of $s_i$ and $s_{i+2}$. By construction, it is possible to get the position of each suffix in the corresponding profile in constant time. Then, we cluster the corresponding profiles of contiguous pairs if they have an LCP value greater than or equal to $\mathcal{L}$ and they are also aligned. This clustering procedure can be done in $\mathcal {O}(k d ^2)$ time.

#### Step 3: Pairs verification

After getting the set of candidate profile pairs, a naïve solution would be to compute the distance for each pair of profiles by comparing them in linear time, i.e., $\mathcal {O}(m)$ time. However, if we compute the LCP array of *s*, we can then perform a sequence of $\mathcal {O}(k)$ RMQ over the LCP array for checking if a pair of profiles is at distance at most *k*. These RMQ over the LCP array correspond to longest common prefix queries between a pair of suffixes of *s*. Since after a linear-time pre-processing over the LCP array, RMQ can be answered in constant time per query [17], we obtain a faster approach for computing the distances. This alternative approach takes $\mathcal {O}(k)$ time to verify each candidate profile pair instead of $\mathcal {O}(m)$ time.

#### Average-case analysis

Algorithm 1 below details the solution based on LCP clusters; and Theorem 1 shows that this algorithm runs in linear time on average using linear space. We rely here on well-known results concerning the linear-time construction of the SA [19, 20] and the LCP array [21], as well as the linear-time pre-processing for the RMQ data structure [18].

**Table 3:** Time and percentage of pairs processed for each method and dataset

Dataset	*k*	Naïve		Binary search		LCP clusters	
		t (s)	Pairs (%)	t (s)	Pairs (%)	t (s)	Pairs (%)
*C. jejuni*	8	108.59	100	0.22	0.06	*0.17*	0.06
	16	109.30	100	0.48	0.32	*0.34*	0.32
	32	108.60	100	3.52	5.45	*2.67*	5.45
	64	*108.60*	100	231.05	99.98	162.36	99.98
*S. enterica*	8	89.85	100	1.04	2.37	*0.95*	2.37
	16	87.26	100	7.16	12.69	*6.73*	12.69
	32	85.36	100	36.29	33.22	*30.76*	33.22
	64	*84.63*	100	254.45	82.44	187.15	82.44
*S. typhi*	89	28.83	100	16.63	91.48	*12.02*	91.48
	178	*28.32*	100	46.98	99.91	32.03	99.91
	890	*30.04*	100	113.57	100	129.14	100
*S. pneumoniae*	8	0.56	100	0.02	0.93	*0.02*	0.93
	16	0.57	100	0.05	1.71	*0.04*	1.71
	32	0.56	100	0.20	4.42	*0.15*	4.42
	64	*0.58*	100	5.63	73.36	5.01	73.36

The minimum time for each row is highlighted in italic

In what follows, $\text {LCP}[i]$, $i>0$, stores the length of the longest common prefix of suffixes $s_{i-1}$ and $s_{i}$ of *s*, and $\text {RMQ}_{\text {LCP}}(i,j)$ returns the index of the smallest element in the subarray $\text {LCP}[i\ldots j]$ in constant time [18]. We rely also on some auxiliary subroutines; let $\mathcal{L}= {\lfloor \tfrac{m}{k+1} \rfloor }$:

- **Aligned**(i) Let $\ell = i\mod m$, i.e., the starting position of the suffix $s_i$ within a profile. Then this subroutine returns $\ell /\mathcal{L}$ if $\ell$ is multiple of $\mathcal{L}$, and $-1$ otherwise.
- **HD**( $p_i$ , $p_j$ , $\ell$) Given two profiles $p_i$ and $p_j$ which share a substring of length $\mathcal{L}$, starting at index $\ell \mathcal{L}$, this subroutine computes the minimum of *k* and the Hamming distance between $p_i$ and $p_j$. This subroutine relies on $\text {RMQ}_{\text {LCP}}$ to find matches between $p_i$ and $p_j$ and, hence, it runs in $\mathcal {O}(k)$ time since it can terminate after *k* mismatches.

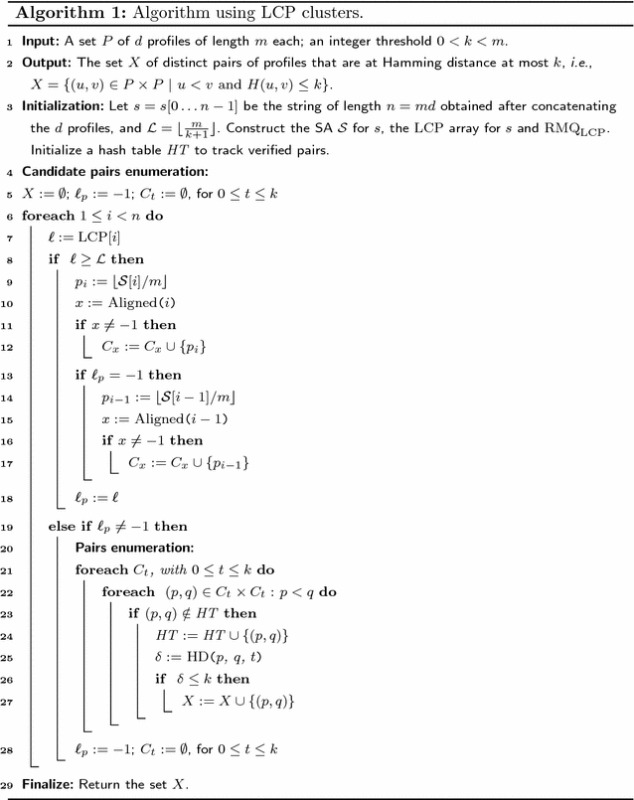

##### **Theorem 1**

*Given d profiles of length m each over an integer alphabet* $\Sigma$ *of size* $\sigma >1$ *with the letters of the profiles being independent and identically distributed random variables uniformly distributed over* $\Sigma,$ *and the maximum Hamming distance* $0< k < m,$ *Algorithm 1 runs in* $\mathcal {O}(m d)$ *average-case time and space if*

$$\begin{aligned} k < \frac{(m-k-1) \cdot \log \sigma }{\log m d}. \end{aligned}$$

##### *Proof*

Let us denote by *s* the string of length *md* obtained after concatenating the *d* profiles. The time and space required for constructing the SA and the LCP arrays for *s* and the RMQ data structure over the LCP array is $\mathcal {O}(md)$.

Let us denote by ${\mathcal B}$ the total number of blocks over *s* and by $\mathcal{L}$ the block length. We set ${\mathcal L}={\lfloor \tfrac{m}{k+1} \rfloor }$ and thus we have that ${\mathcal B} = {d \lfloor \tfrac{m}{\mathcal{L}}\rfloor }$. Let us also denote by *C* a maximal set of indices over *x* satisfying the following:

1. The length of the longest common prefix between any two suffixes of *s* starting at these indices is at least $\mathcal{L}$;
2. both of these suffixes start at the starting position of a block;
3. and both indices correspond to the starting position of the *i*th block in their profiles.

This can be done in $\mathcal {O}(md)$ time using the LCP array (lines 7–17). Processing all such sets *C* (lines 21–27) requires total time

$$\begin{aligned} \textsf {PROC}_{i,j} \times {Pairs} \end{aligned}$$

where $\textsf {PROC}_{i,j}$ is the time required to process a pair *i*, *j* of elements of a set *C*, and *Pairs* is the sum of $|C|^2$ over all such sets *C*. We have that $\textsf {PROC}_{i,j}=\mathcal {O}(k)$ by using RMQ over the LCP array. Additionally, by the stated assumption on the *d* profiles, the expected value for *Pairs* is no more than $\frac{{\mathcal B}d}{\sigma ^\mathcal{L}}$: we have ${\mathcal B}$ blocks in total and each block can only match at most *d* other blocks by the conditions above. Hence, the algorithm requires on average the following running time

$$\begin{aligned} \mathcal {O}\left(md + k \cdot \frac{{\mathcal B}d}{\sigma ^{\mathcal{L}}}\right). \end{aligned}$$

Let us analyze this further to obtain the relevant condition on *k*. We have the following:

$$\begin{aligned} k \cdot \frac{{\mathcal B}d}{\sigma ^{\mathcal{L}}} = {\frac{k \cdot \lfloor \tfrac{m}{\lfloor m/(k+1) \rfloor}\rfloor \cdot d^2}{\sigma ^ {\lfloor \frac{m}{k+1}\rfloor }}} \le {\frac{k\cdot \left(\tfrac{m}{ \lfloor m/(k+1) \rfloor }\right) \cdot d^2}{\sigma ^ {\frac{m}{k+1}-1}}}. \end{aligned}$$

Since $0< k < m$ by hypothesis, we have the following:

$$\begin{aligned} {\frac{k \cdot \left(\tfrac{m}{ \lfloor m/(k+1) \rfloor }\right) \cdot d^2}{\sigma ^ {\frac{m}{k+1}-1}}} \le {\frac{(md)^2}{\sigma ^ {\frac{m}{k+1}-1}}}. \end{aligned}$$

By some simple rearrangements we have that:

$$\begin{aligned} {\frac{(md)^2}{\sigma ^ {\frac{m}{k+1}-1}}} = {\frac{(md)^2}{(md)^ {\frac{\log \sigma }{\log md} \left(\frac{m}{k+1}-1\right)}}}= (md)^{2-\frac{(m-k-1) \log \sigma }{(k+1) \log md}}. \end{aligned}$$

Consequently, in the case when

$$\begin{aligned} k < \frac{(m-k-1) \cdot \log \sigma }{\log md} \end{aligned}$$

the algorithm requires $\mathcal {O}(md)$ time on average. The extra space usage is clearly $\mathcal {O}(md)$. $\square$

### Use case 1: goeBURST algorithm

The distance matrix computation is a main step in distance-based methods for phylogenetic inference. This step dominates the running time of most methods, taking $\Theta (m d^2)$ time, for *d* sequences of length *m*, since it must compute the distance among all sequence pairs. But for some methods, or when we are only interested in local phylogenies for sequences or profiles of interest, one does not need to know all pairwise distances for reconstructing a phylogenetic tree. The problem addressed in this article was motivated by the goeBURST algorithm [10], our use case 1. goeBURST is one of such methods for which one must know only the pairs of sequences that are at Hamming distance at most *k*. The solution proposed here can however be extended to other distance-based phylogenetic inference methods, that rely directly or indirectly on Hamming distance computations. Note that most methods either consider the Hamming distance or its correction accordingly to some formula based on some model of evolution [2, 4]. In both cases we must start by computing the Hamming distance among sequences, but not necessarily all of them [6].

The underlying model of goeBURST is as follows: a given genotype increases in frequency in the population as a consequence of a fitness advantage or of random genetic drift, becoming a founder clone in the population; and this increase is accompanied by a gradual diversification of that genotype, by mutation and recombination, forming a cluster of phylogenetic closely-related strains. This diversification of the “founding” genotype is reflected in the appearance of genetic profiles differing only in one housekeeping gene sequence from this genotype—single locus variants (SLVs). Further diversification of those SLVs will result in the appearance of variations of the original genotype with more than one difference in the allelic profile, e.g., double and triple locus variants (DLVs and TLVs).

The problem solved by goeBURST can be stated as a graphic matroid optimization problem and, hence, it follows a classic greedy approach [22]. Given the maximum Hamming distance *k*, we can define a graph $G = (V, E)$, where $V = P$ (set of profiles) and $E= \{ (u,v) \in V^{2} \ |\ H(u,v) \le k \}$. The main goal of goeBURST is then to compute a minimum spanning forest for *G* taking into account the distance *H* and a total order on links. It starts with a forest of singleton trees (each sequence/profile is a tree). Then it constructs the optimal forest by adding links connecting profiles in different trees in increasing order accordingly to the total order, similarly to what is done in the Kruskal’s algorithm [23]. In the current implementation, a total order for links is implicitly defined based on the distance between sequences, on the number of SLVs, DLVs, TLVs, on the occurrence frequency of sequences, and on the assigned sequence identifier. With this total order, the construction of the tree consists of building a minimum spanning forest in a graph [23], where each sequence is a node and the link weights are defined by the total order. By construction, the pairs at distance $\delta$ will be joined before the pairs at distance $\delta +1$.

### Use case 2: querying typing databases

A related problem is querying typing databases for similar typing profiles. Given a set *P* of *d* profiles of length *m* each, a profile *u* not necessarily in *P* but with the same length *m* as those in *P*, and *k* such that $0<k<m$, the problem is to find all profiles $v\in P$ such that $H(u,v)\le k$. One may be also interested on local phylogenetic patterns, but those can be inferred from found profiles using for instance the goeBURST algorithm.

Once we define the value for *k*, we can address this problem as follows. We index all *d* profiles in the database as before in linear time $\mathcal {O}(m d)$, and given a query profile *u*, we enumerate all candidate profiles *v*. We then verify as before all candidate pairs and we return only those satisfying $H(u,v)\le k$.

For indexing set *P*, we make use of the suffix tree data structure. The *suffix tree* $\mathcal {T}(x)$ of a string *x* is a compact trie representing all suffixes of *x*. It is known that the suffix tree of a string of length *n*, over an integer alphabet, can be computed in time and space $\mathcal {O}(n)$ [24]. For integer alphabets, in order to access the children of an explicit node of the suffix tree by the first letter of their edge label in $\mathcal {O}(1)$ time, we make use of *perfect hashing* [25].

By using the suffix tree we find candidate matches through forward search: spelling blocks of *u* from the root. Specifically, given the $k+1$ non-overlapping blocks of length $\mathcal{L}={\lfloor \tfrac{m}{k+1} \rfloor }$ of *u*, we search (without reporting) for each one of them in $\mathcal {O}(\mathcal {L})$ time. Since we have $k+1$ blocks, it takes $\mathcal {O}(k \mathcal {L}) = \mathcal {O}(m)$ time to search for all $k+1$ blocks of *u*. Finally, we can verify and report all candidate profiles $v\in P$ as detailed in Algorithm 2.
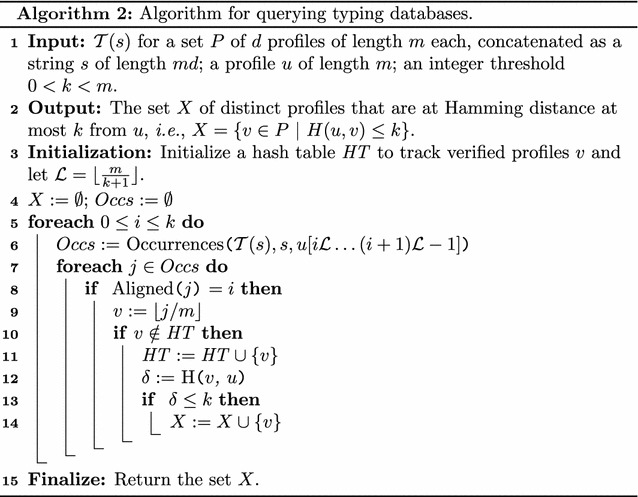


Although, in the worst case, Algorithm 2 runs in time $\mathcal {O}(m d + m\log md)$, as we may have *d* matches at most, we can prove a similar average case as in Theorem 1.

#### **Theorem 2**

Given a profile *u* and a set of *d* profiles of length *m* each, all over an integer alphabet $\Sigma$ of size $\sigma >1$, with the letters of the profiles being independent and identically distributed random variables uniformly distributed over $\Sigma$, the $\mathcal {T}(s)$ for the string *s* of length *md* obtained after concatenating the *d* profiles, and the maximum Hamming distance $0< k < m$, Algorithm 2 runs in $\mathcal {O}(m)$ average-case time if

$$\begin{aligned} k < \frac{(m-k-1) \cdot \log \sigma }{\log m d}. \end{aligned}$$

#### *Proof*

Let us denote by $\mathcal{B}$ the total number of blocks over *s* and by $\mathcal{L}$ the block length. We set $\mathcal{L}={\lfloor \tfrac{m}{k+1} \rfloor }$ and thus we have that ${\mathcal B} = {d \lfloor \tfrac{m}{{\mathcal L}}\rfloor }$.

By the stated assumption on the profiles, the expected value for the number of profiles matching *u* is no more than $\frac{\mathcal B}{\sigma ^{\mathcal L}}$: we have $\mathcal{B}$ blocks in total and each block can only match at most one other block in *u* (since *they must be aligned*; line 8).

Moreover, since we are not relying on the LCP array in this case (profile *u* is not indexed), the verification step (line 12) takes $\mathcal {O}(m)$ time using letter comparisons. Hence, the algorithm requires on average the following running time

$$\begin{aligned} \mathcal {O}(m + m \cdot \frac{\mathcal{B}}{\sigma ^{\mathcal{L}}}). \end{aligned}$$

Let us analyze this further to obtain the relevant condition on *k*. We have the following:

$$\begin{aligned} m \cdot \frac{\mathcal{B}}{\sigma ^{\mathcal{L}}} = {\frac{m \cdot \lfloor \tfrac{m}{\lfloor m/(k+1) \rfloor }\rfloor \cdot d}{\sigma ^ {\lfloor \frac{m}{k+1}\rfloor }}} \le {\frac{m\cdot (\tfrac{m}{ \lfloor m/(k+1) \rfloor }) \cdot d}{\sigma ^ {\frac{m}{k+1}-1}}} \le {\frac{m^2 d}{\sigma ^ {\frac{m}{k+1}-1}}}. \end{aligned}$$

By some simple rearrangements we have that:

$$\begin{aligned} {\frac{m^2 d}{\sigma ^ {\frac{m}{k+1}-1}}} = {\frac{m^2 d}{(md)^ {\frac{\log \sigma }{\log md} (\frac{m}{k+1}-1)}}}= m (md)^{1-\frac{(m-k-1) \log \sigma }{(k+1) \log md}}.\end{aligned}$$

Consequently, in the case when

$$k < \frac{(m-k-1) \cdot \log \sigma }{\log m d}$$

the algorithm requires $\mathcal {O}(m)$ time on average.$\square$

This algorithm was implemented using a suffix array and then integrated in INNUENDO Platform, which is publicly available [24]. The INNUENDO Platform is an infrastructure that provides the required framework for data analyses from bacterial raw reads sequencing data quality insurance to the integration of epidemiological data and visualization. As such, rapid methods for classification and search for closely related strains are a necessity for quick navigation through the platform database entries. More information about the project can be found at its website [25].

As a starting point and for the purpose of this study, a subset of 2312 wgMLST profiles of *Escherichia coli* retrieved from Enterobase [13] were included in the INNUENDO database as well as their ancillary data and predefined core-genome cluster classification. Two tab-separated files containing the wgMLST and cgMLST profiles for the *E. coli* strains were also created to allow storing information on the currently available profiles and for updating with profiles that will become available upon the platform analyses.

One of two index files are used depending on the type of search we want to perform: classification or search for *k*-closest. The cgMLST index file is used for strain classification, which relies on a nomenclature designed for the cgMLST profiles. As such, and since a pre-classification was performed on the database of *E. coli* strains, we continued using it for comparison purposes. However, when searching for the *k*-closest profiles, we take into consideration all targets available in the wgMLST profiles using the wgMLST index file for a higher discriminatory power.

Each time a new profile is generated from the platform, it requires classification. The INNUENDO Platform performs the classification step based on the approach described in our "Use case 2: querying typing databases" with a given maximum of *k* differences over core genes. It uses the cgMLST index file for the search since the classification is constructed based on those number of loci. If the method returns at least one match, it classifies the new profile with the classification of the closest. If not, a new classification is assigned. A new entry is then added to the INNUENDO database as well as to the cgMLST and wgMLST profiles files and the index files are updated.

In the case of the search for the *k*-closest, it is useful to define the input data for visualization methods according to a defined number of differences on close strains. For each profile used as input for the search, the method searches for the *k*-closest strains considering at most *k* differences among all wgMLST loci. Since duplicate matches can occur between the profiles used for each search, the final file used as input for the visualization methods is the intersection of the results of the *k*-closest profiles between each input strain. The set of strain identifiers are then used to query the INNUENDO database to get the profiles and ancillary data to be sent to PHYLOViZ Online [26] for further analysis, namely with the goeBURST algorithm.

The drawback of using this method for classification and search is the need for rebuilding the index each time there is a new profile, which will depend on the number of profile entries on the database. Nevertheless, the number of updates is rather smaller compared to the number of queries and the index can be build in the background, with search functionalities still using the old index during the process. In our implementation, the index and related data structures are serialized in secondary memory and they are accessed by mapping them into memory. The implementation of the underlying tool is made publicly available [27].

The above described approaches in combination with the features offered by the INNUENDO Platform allow microbiologists to quickly and efficiently search for strains close to their strain of interest, allowing a more targeted, focused and simple visualization of results.

## Experimental evaluation

We evaluated the proposed approach to compute the pairs of profiles at distance at most *k* using both real and synthetic datasets. We used real datasets obtained through different typing schemas, namely whole-genome multi-locus sequence typing (wgMLST) data, core-genome multi-locus sequence typing (cgMLST) data, and single-nucleotide polymorphism (SNP) data. Table 2 summarizes the real datasets used. We should note that wgMLST and cgMLST datasets contain sequences of integers, where each column corresponds to a locus and different values in the same column denote different alleles. Synthetic datasets comprise sets of binary sequences of variable length, uniformly sampled, allowing us to validate our theoretical findings.

We implemented both versions described above in the C programming language: one based on binary search over the SA; and another one based on finding clusters in the LCP array. Since allelic profiles can be either string of letters or sequences of integers, we relied on libdivsufsort library [28] and qsufsort code [29, 30], respectively. For RMQ over the LCP array, we implemented a fast well-known solution that uses constant time per query and linearithmic space for pre-processing [17].

All tests were conducted on a machine running Linux, with an Intel(R) Xeon(R) CPU E5-2630 v3 @ 2.40 GHz (8 cores, cache 32 KB/4096 KB) and with 32 GB of RAM. All binaries where produced using GCC 5.3 with full optimization enabled.

### Synthetic datasets

We first present results with synthetic data for different values of *d*, *m* and *k*. All synthetic sequences are binary sequences uniformly sampled. Results presented in this section were averaged over ten runs and for five different sets of synthetic data.

The bound proved in Theorem 1 was verified in practice. For *k* satisfying the conditions in Theorem 1, the running time of our implementation grows almost linearly with *n*, the size of the input. We can observe in Fig. 1 a growth slightly above linear. Since we included the time for constructing the SA, the LCP array and the RMQ data structure, with the last one in linearithmic time, that was expected.

We also tested our method for values of *k* exceeding the bound shown in Theorem 1. For $d=m=4096$ and a binary alphabet, the bound for *k* given in Theorem 1 is no more than $\lfloor m/(2\log m)\rfloor =170$. For *k* above this bound we expect that proposed approaches are no longer competitive with the naïve approach. As shown in Fig. 2, for $k>250$ and $k>270$ respectively, both limits above the predicted bound, the running time for both computing pairwise distances by finding lower and higher bounds in the SA, and by processing LCP based clusters, becomes slower than the running time of the naïve approach.

In Fig. 3 we have the running time as a function of the number *d* of profiles, for different values of *m* and for *k* satisfying the bound given in Theorem 1. The running time for the naïve approach grows quadratically with *d*, while it grows linearly for both computing pairwise distances by finding lower and higher bounds in the SA, and by processing LCP based clusters. Hence, for synthetic data, as described by Theorem 1, the result holds.

### Real datasets

For each dataset in Table 2, we ranged the threshold *k* accordingly and compared the approaches discussed in "Methods" section with the naïve approach that computes the distance for all sequence pairs. Results are provided in Table 3.

In most cases, the approach based on the LCP clusters is the fastest up to two orders of magnitude compared to the naïve approach. As expected, in the case when data are not uniformly random, our method works reasonably well for smaller values of *k* than the ones implied by the bound in Theorem 1. As an example, the upper bound on *k* for *C. jejuni* would be around 200, but the running time for the naïve approach is already better for $k=64$. We should note however that the number of candidate profile pairs at Hamming distance at most *k* is much higher than the expected number when data are uniformly random. This tells us that we can design a simple hybrid scheme that chooses a strategy (naïve or the proposed method) depending on the nature of the input data. It seems also to point out clustering effects on profile dissimilarities, which we may exploit to improve our results. We leave both tasks as future work for the full version of this article.

We incorporated the approach based on finding lower and higher bounds in the SA in the implementation of goeBURST algorithm, discussed in "Methods" section. We did not incorporate the approach based on the LCP clusters as the running time did not improve much as observed above. Since running times are similar to those reported in Table 3, we discuss only the running time for *C. jejuni*. We need only to index the input once. We can then use the index in the different stages of the algorithm and for different values of *k*. In the particular case of goeBURST, we use the index twice: once for computing the number of neighbors at a given distance, used for untying links according to the total order discussed in the description of goeBURST algorithm in methods section, and a second time for enumerating pairs at distance below a given threshold. Note that the goeBURST algorithm does not aim to link all nodes, but to identify clonal complexes (or connected components) for a given threshold on the distance among profiles [10]. In the case of *C. jejuni* dataset, and for $k=52$, the running time is around 36 s, while the naïve approach takes around 115 s, yielding a threefold speedup. In this case we get several connected components, i.e., several trees, connecting the most similar profiles. We provide the tree for the largest component in Fig. 4, where each node represents a profile. The nodes are colored according to one of the loci for which profiles in this cluster differ. Note that this tree is optimal with respect to the criterion used by the goeBURST algorithm, not being affected by the threshold on the distance. In fact, since this problem is a graphic matroid, the trees found for a given threshold will be always subtrees of the trees found for larger thresholds [22]. Comparing this tree with other inference methods is beyond the scope of this article; the focus here was on the faster computation of an optimal tree under this model.

In many studies, the computation of trees based on pairwise distances below a given threshold, usually small compared with the total number of loci, combined with ancillary data, such as antibiotic resistance and host information, allows microbiologists to uncover evolution patterns and study the mechanisms underlying the transmission of infectious diseases [31].

## Conclusions

Most distance-based phylogenetic inference methods rely directly or indirectly on Hamming distance computations. The computation of a distance matrix is a common first step for such methods, taking $\Theta (m d^2)$ time in general, with *d* being the number of sequences or profiles and *m* the length of each sequence or profile. For large-scale datasets this running time may be problematic; however, for some methods, we can avoid to compute all-pairs distances [6].

We addressed this problem when only a truncated distance matrix is needed, i.e., one needs to know only which pairs are at Hamming distance at most *k*. This problem was motivated by the goeBURST algorithm [10], which relies on a truncated distance matrix by construction. Both the problem and techniques discussed here are related to average-case approximate string matching [32, 33]. We proposed here an average-case linear-time and linear-space algorithm to compute the pairs of sequences or profiles that are at Hamming distance at most *k*, when $k < \frac{(m-k-1) \cdot \log \sigma }{\log m d}$, where $\sigma$ is the size of the alphabet. We integrated our solution in goeBURST demonstrating its effectiveness using both real and synthetic datasets.

We must note however that our analysis holds for uniformly random sequences and, hence, as observed with real data, the presented bound may be optimistic. It is thus interesting to investigate how to address this problem taking into account local conserved regions within sequences. Moreover, it might be interesting to consider in the analysis null models such as those used to evaluate the accuracy of distance-based phylogenetic inference methods [4].

The proposed approach is particularly useful when one is interested in local phylogenies, i.e., local patterns of evolution, such as searching for similar sequences or profiles in large typing databases, as in our "Use case 2: querying typing databases". In this case we do not need to construct full phylogenetic trees, with tens of thousands of taxa. We can focus our search on the most similar sequences or profiles, within a given threshold *k*. There are however some issues to be solved in this scenario, namely, dynamic updating of the data structures used in our algorithm. Note that after querying a database, if new sequences or profiles are identified, then we should be able to add them while keeping our data structures updated. Although more complex and dynamic data structures are known, a technique proposed recently for adding dynamism to otherwise static data structures can be useful to address this issue [34]. This and other challenges raised above are left as future work.
